# Community-Based Noncommunicable Disease Care for Syrian Refugees in Lebanon

**DOI:** 10.9745/GHSP-D-17-00043

**Published:** 2017-09-27

**Authors:** Stephen Sethi, Rebecka Jonsson, Rony Skaff, Frank Tyler

**Affiliations:** a Medical Teams International, Portland, Oregon, USA.; b Medical Teams International, Zahle, Lebanon.

## Abstract

In the sixth year of the Syrian conflict, 11 million people have been displaced, including more than 1.1 million seeking refuge in Lebanon. Prior to the crisis, noncommunicable diseases (NCDs) accounted for 80% of all deaths in Syria, and the underlying health behaviors such as tobacco use, obesity, and physical inactivity are still prevalent among Syrian refugees in Lebanon. Humanitarian agencies initially responded to the acute health care needs of refugees by delivering services to informal settlements via mobile medical clinics. As the crisis has become more protracted, humanitarian response plans have shifted their focus to strengthening local health systems in order to better address the needs of both the host and refugee populations. To that end, we identified gaps in care for NCDs and launched a program to deliver chronic disease care for refugees. Based on a participatory needs assessment and community surveys, and building on the success of community health programs in other contexts, we developed a network of 500 refugee outreach volunteers who are supported with training, supervision, and materials to facilitate health promotion and disease control for community members, target NCDs and other priority conditions, and make referrals to a primary health care center for subsidized care. This model demonstrates that volunteer refugee health workers can implement community-based primary health activities in a complex humanitarian emergency.

## Abstract (precis)

The high prevalence of noncommunicable diseases (NCDs) among Syrian refugees in Lebanon required a shift in the humanitarian response, from direct care provided through mobile medical clinics to community-based primary health care and health promotion provided through trained refugee outreach volunteers (ROVs). During the first 2 months after training, these ROVs conducted 753 blood pressure monitoring visits and 657 blood glucose checks; monitored medication adherence among 387 patients with NCDs; referred 293 refugees to the local primary health care facility for additional care; and provided 346 targeted health education messages.

## CONTEXT

### Global and Regional Refugee Trends

The world is currently experiencing the highest levels of displacement on record, with 65.3 million people forced from their homes by the end of 2015, including nearly 21.3 million refugees, over half of whom are children.^1^ More than half of all refugees have fled from only 3 countries: Syria, Afghanistan, and Somalia. Syria's civil war in particular is considered the worst complex humanitarian emergency of our time, with more than 11 million people displaced in the sixth year of the conflict. According to the United Nations High Commissioner for Refugees (UNHCR), which is leading the regional emergency response, 4.8 million Syrians have fled to Turkey, Lebanon, Jordan, Egypt, and Iraq; another 6.6 million are internally displaced within Syria; and about 1 million have requested asylum in Europe.^1^ As a result, the refugee crisis has created tremendous strains on host countries such as Lebanon, where more than 1.1 million Syrian refugees account for 25% of the entire population.

The refugee crisis has created tremendous strains on host countries such as Lebanon, where more than 1.1 million Syrian refugees account for 25% of the entire population.

After 6 years of conflict in Syria, the protracted nature of displacement has substantially strained the health resources of host countries and even international organizations. For the regional response to the Syrian crisis in 2016, UNHCR intended to support 4.2 million primary health consultations, 308,000 referrals for secondary or tertiary care, and expand access at 300 health facilities for 4.8 million refugees; however, funding at the end of 2016 only reached 56% of the target.^2^ As a consequence, national health systems lack sufficient delivery capacity to support a significantly larger population, consequently impacting the health needs of the host populations as well. In Lebanon, this is further complicated by the lack of universal health care and a system dominated by private health service providers,^3^^,^^4^ in which private spending accounts for 52% of total health expenditures.^5^ The result was that some vulnerable low-income host Lebanese communities were initially not eligible for the same health subsidies as refugees. To reduce those disparities, recent crisis response plans have emphasized integration of services through public primary health care (PHC) centers.

### Noncommunicable Diseases Among Refugees

The massive growth in migration from and to more developed countries has altered the balance of health issues faced by refugees and internally displaced persons (IDPs). While acute infectious diseases remain a priority in some refugee contexts, noncommunicable diseases (NCDs) make up a greater share of the burden of those displaced from conflicts in Iraq, Syria, and Ukraine. NCDs accounted for 19% to 46% of mortality in the top 5 source countries for refugees in 2015.^6^ This parallels a global trend in which deaths due to infectious diseases have declined, life expectancy has increased, unhealthy lifestyle behaviors have spread to developing economies, and the overall proportion of deaths due to NCDs has risen to more than 50%. Already, more than 80% of deaths from NCDs occur in low- and middle-income countries.^7^^,^^8^ The trend toward a higher prevalence of chronic diseases among refugees is further reinforced by flows into developed countries where lifestyle risk factors, such as tobacco and alcohol use, poor diet, and inactivity, favor the development of diabetes, cardiovascular disease, cancers, and chronic lung diseases. For the 6.7 million refugees worldwide who have been displaced for more than 5 years,^9^ the profile of health needs has begun to more closely reflect that of their host country's population, as behaviors adapt to the local environments over time.

For the 6.7 million refugees worldwide who have been displaced for more than 5 years, the profile of health needs has begun to more closely reflect that of their host country's population.

In the case of Syrian refugees in Jordan, Lebanon, and Turkey, in 2010 (before the influx of refugees), NCDs accounted for 77% to 90% of mortality in the region, while the rate in Syria was estimated at 80%.^10^ In Lebanon, 43% of men and 27% of women smoke tobacco,^11^ per capita alcohol consumption is 2.4 liters per year,^12^ 37% are overweight, 28% are obese, and 68.7% are physically inactive.^13^ Prior to the civil war in Syria, 48% of men and about 9% of women smoked, per capita alcohol use was 1.2 liters per year, 27% were obese, and 25% had hypertension.^6^ Thus, Syrians who were already highly affected by NCDs before displacement are now living in environments that continue to support unhealthy behaviors and are likely to provide less support to mitigating or managing those diseases.

During rapid-onset emergencies, health action has necessarily focused on acute conditions. However, in the past several years, the humanitarian community has also recognized the importance of caring for displaced people with NCDs. According to the United Nations Interagency Task Force on NCDs, emergencies interrupt the normal coordinated systems of care for chronic diseases, which exist to prevent, detect, monitor, treat, and manage these diseases and their complications.^14^ In emergency settings, people living with NCDs may experience complications or challenges, such as acute exacerbations of heart and lung conditions, loss of access to medications, physical strains of displacement on the elderly and disabled, and destruction of infrastructure and resources, such as dialysis equipment. These issues are gaining consideration, with peer-reviewed journals publishing articles on identified research gaps on effective interventions^15^^,^^16^ and recent symposia focusing on NCDs in humanitarian settings.^17^ This new attention builds on high-level advocacy for NCDs in developing countries, including the World Health Organization (WHO)'s *Global Action Plan for the Prevention and Control of NCDs*^18^ and *Package of Essential Noncommunicable (PEN) Disease Interventions for Primary Health Care in Low-Resource Settings*.^19^

### Community-Based Primary Health Care

Constrained health facility-based resources limit the scale at which NCD care can be provided in countries that are still grappling simultaneously with high rates of communicable diseases and malnutrition.^20^ Community health models have a successful history of task shifting the delivery of care out of facilities enabled, in part, by WHO's endorsement of community-based primary health care in the Alma-Ata Declaration of 1978.^22^ There are an estimated 1.3 million community health workers (CHWs) worldwide.^21^ Large-scale CHW programs have proven the value of mobilizing communities to extend the delivery of preventive and curative services—particularly for maternal and child health—by filling health gaps not met by facilities alone.^22^

Large-scale CHW programs have proven the value of mobilizing communities to extend the delivery of preventive and curative services by filling health gaps not met by facilities alone.

Community outreach programs have been expanded to include NCDs as well, with evidence that CHWs can accurately assess and manage cardiovascular risk,^23^^,^^24^ reduce the onset of hypertension through behavior change messages,^25^ improve control of hypertension and diabetes,^26^ conduct health screenings, provide referrals to health facilities, monitor patients, and track health outcomes.^27^ Some efforts have also recently been made to mobilize refugees as CHWs, through training and support, to provide basic maternal, newborn, and child health services. A recent review of the published evidence on the impact of refugee CHWs found positive population health outcomes across a variety of interventions, including reproductive, maternal, and child health.^28^ The demonstrated value of a trained community-based workforce led the Global Health Workforce Alliance to promote expanding CHW programs for disaster risk reduction, preparedness, emergency response, and recovery.^29^ Given the evidence for successful community health programs in NCDs and experience with mobilizing refugees as health workers, we describe our program for integrating these approaches in the context of the Syrian refugee crisis in Lebanon.

## PROGRAM DESCRIPTION

### Mobile Service Delivery Phase

During the early response to the refugee crisis in Lebanon, health agencies, including ours at Medical Teams International, focused on providing care through mobile medical clinics to patients with acute medical needs who had been otherwise unable to access care due to financial constraints, lack of mobility, and residency issues. However, research conducted in 2014 found that most Syrian refugee (50.4%) and Lebanese (60.2%) households have a member with an NCD,^30^ and almost 83% of refugees with an NCD sought care for their disease, with 58% going to a public PHC facility for their condition. In 2013, our organization conducted an assessment in collaboration with the UNHCR Bekaa Health Working Group, and identified significant gaps in NCDs and dental health services for refugees. In 2014, we launched an NCD and children's dental project in the Bekaa Valley, which served 32 informal settlements (ISs) through mobile medical clinics providing clinical consultations, medications, disease monitoring, health education, and referrals to supported PHC facilities for diagnostic tests and children's dental care. In 2015, we expanded the project area to 120 ISs, and started to refer some Syrian refugees to nearby PHC facilities that had begun to receive support from other health agencies. During the 2 years of the mobile medical project, our local clinicians managed the care of 2,000 NCD patients, with more than 18,000 consultations; delivered almost 54,000 prescription medications; screened 10,500 children for dental problems; and facilitated acute dental care for 1,450 children (Box).

During the 2 years of the mobile medical project in Lebanon, our local clinicians managed the care of 2,000 NCD patients from 120 informal settlements.

BOX **Mobile Clinic Patient Characteristics of Syrian Refugees in Lebanon, 2014–2016**

- 2,000 NCD patients
  - Median age: 53
  - Female to male ratio: 1.27 to 1.00
  - Average systolic blood pressure: 140.7 mmHg
  - Average random capillary blood glucose: 181
  - Sedentary lifestyle: 66%
  - Obese: 39%
  - Smokers: 37.5%
  - Consume excess salt: 32%
- 18,274 patient consultations
  - Dyslipidemia: 13.8%
  - Hypertension: 76%
  - Type 2 diabetes: 39%
  - Asthma/COPD: 10%
  - Cardiovascular disease: 7%
  - Type 1 diabetes: 1.4%
- 53,785 prescriptions
  - Adherence to medication regimen most or all of the time: 95%

Abbreviations: COPD, chronic obstructive pulmonary disease; NCD, noncommunicable disease.

To further investigate the effectiveness of the ongoing NCD project and potential gaps and barriers, in 2015, we conducted a random survey, using Lot Quality Assurance Sampling,^31^ of 320 NCD patients attending 10 mobile medical clinics, and analyzed the data using EpiInfo 7 (U.S. Centers for Disease Control and Prevention, Atlanta, GA, USA). The results showed evidence of strong clinical NCD management but ongoing gaps in the underlying unhealthy behaviors and disease knowledge. For example, about 80% of the patients said they took their medications all of the time; about 97% of patients with hypertension reported checking their blood pressure monthly; and approximately 96% of patients with diabetes indicated they get their blood glucose checked at least 1 time each month. But 34% of patients said they currently smoked, 21% of patients with hypertension said they add salt to their food most or all the time, and only 64% of patients with diabetes said that eating fewer sweets would help control their disease (Table 1).

**TABLE 1.** Mobile Clinic Noncommunicable Disesase Patient Survey of Syrian Refugees in Lebanon, 2015 (N=320)

	%	95% CI
**Demographics**		
Age, years, mean (SD)	54.6	(11.4)
Female	59.4	(53.8, 64.8)
**Disease control among all patients**		
Adhere to medications “all of the time”	79.9	(74.95, 84.1)
Follow a diet to control their hypertension or diabetes	70.7	(64.8, 76.2)
Currently smoking	34.4	(29.2, 40.1)
Have reduced or quit smoking	13.0	(9.5, 17.4)
**Patients with hypertension (n=227)**		
Check their blood pressure monthly	97.4	(94.3, 99.0)
Add salt to food most or all of the time	21.2	(16.0, 27.0)
Eat salty processed food daily	10.9	(7.0, 15.9)
Eat salty processed food weekly	24.6	(19.0, 31.0)
**Patients with diabetes (n=114)**		
Get at least 1 glucose check monthly	95.6	(90.1, 98.6)
State that taking medication will help to control their disease	72.8	(63.7, 80.7)
State that eating fewer sweets, candies, and pastries will help to control their disease	64.0	(54.5, 72.8)
State that avoiding sugar in tea or coffee will help to control their disease	70.2	(60.9, 78.4)
State that weight loss could improve their disease control	0.0	(0.0, 0.0)
**Patients with chronic lung disease (n=56)**		
Have heard messages about their condition	85.7	(73.8, 93.6)
Know that one type of asthma/COPD medication is for prevention	55.4	(41.5, 68.7)
Know that one type of asthma/COPD medication is for rescue	64.3	(50.4, 76.6)

Abbreviations: CI, confidence interval; COPD, chronic obstructive pulmonary disease; SD, standard deviation.

All data reported as % (95% CI) unless otherwise specified.

With this evidence, we then organized a community-based needs assessment as a step toward establishing a community health program. Using participatory learning and assessment tools,^32^ a representative sample of 117 refugees from 15 ISs was consulted on their perceived health priorities. We discovered that their main health concerns, in order of importance, were NCDs, acute respiratory illnesses, skin infections, arthritis, gastrointestinal problems, vaginal infections, urinary problems, diarrheal illnesses, tuberculosis, anemia, cancer, eye infections, and other less common conditions. To verify that these health conditions were important to the wider refugee community and to establish a baseline of health indicators, we conducted a 30-cluster household survey randomly sampled on probability proportionate to size of IS.^33^ Standardized Knowledge, Practices, and Coverage (KPC) questionnaires^34^ were used in parallel interviews of 300 adults and 300 mothers; the survey of adults covered issues of prevalence of NCDs and related risk factors, access to, use of, and satisfaction with PHC facilities, while the survey of mothers covered issues related to breastfeeding, child vaccination, and experience of episodes of diarrhea and respiratory infections among their children. The results were analyzed with EpiInfo 7 and are summarized in Table 2 and Table 3. The results confirmed that most refugees had access to a PHC facility at a reasonable cost and quality, but significant gaps in knowledge and behaviors and high rates of childhood diseases still remained.

**Figure:**
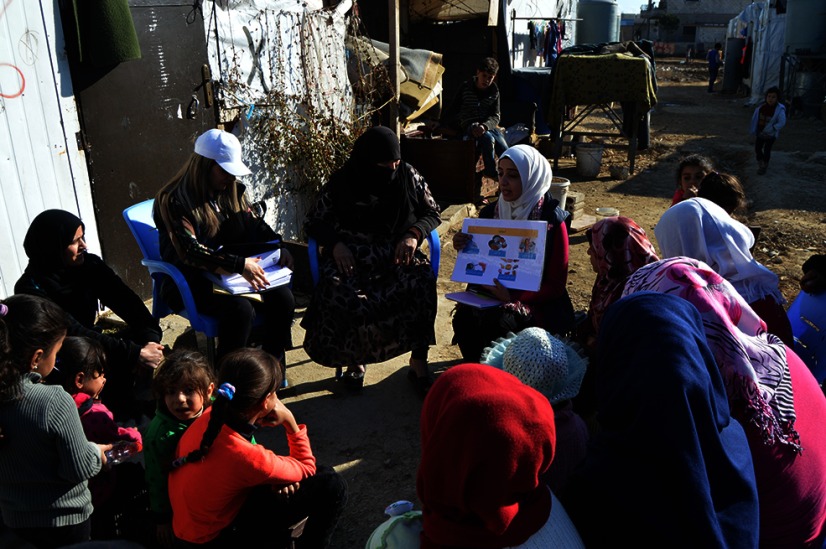
A community health promoter trains refugee outreach volunteers to facilitate health promotion and noncommunicable disease control in their communities.

**TABLE 2.** Knowledge, Practice, and Coverage Survey of Adult Syrian Refugees in Lebanon, 2016 (N=300)

	%	95% CI
Age, years, mean (SD)	36.5	(12.9)
Have diabetes, high blood pressure, heart disease, asthma, or emphysema	22.8	(17.87, 27.20)
**Access to a PHC facility**		
Have been to a PHC facility	76.8	(71.60, 81.10)
Have been to a PHC facility within the last month	25.0	(19.74, 31.35)
Barriers to seeking care at a PHC facility		
Medical costs	17.0	(9.18, 28.03)
Lack of transport	11.0	(5.07, 21.28)
Legal reasons	4.0	(0.89, 12.02)
Lack of time	4.0	(0.89, 12.02)
Did not have a medical need to go to a PHC facility	47.0	(35.09, 59.45)
**Transportation to a PHC facility**^a^		
Transportation method		
Taxi	44.0	(37.57, 50.97)
Walking	36.0	(30.01, 42.84)
Bus	13.0	(9.02, 18.17)
Cost of transport, LBP, mean (SD)	2,000	(282)
Time to get to the PHC facility, minutes, mean (SD)	21.9	(17.2)
**Services and costs at a PHC facility**		
Medical cost of a PHC visit, LBP, mean (SD)	3,000	(833)
Diagnostic tests ordered	25.0	(19.26, 30.76)
Cost of tests ordered, LBP, mean (SD)	20,000	(4,386)
Received a prescription	78.0	(71.89, 83.30)
Able to get the medication at the time of their visit	34.5	(27.41, 42.14)
Cost of medical care including medications, LBP, mean (SD)	7,000	(2,886)
**Reasons for visiting PHC facility**		
Acute disease	45.0	(38.42, 51.67)
Chronic disease	14.0	(9.76, 19.15)
Antenatal care	14.0	(9.76, 19.15)
Well-child visit	10.0	(9.76, 19.15)
Dental care	23.0	(17.45, 28.69)
**Satisfaction with services at PHC facility**		
Describe the care as either good or very good	67.8	(61.37, 73.82)
Feel the care could be improved	73.0	(66.54, 78.47)
Main concerns		
Respect	26.5	(19.97, 33.9)
Wait times	33.0	(26.03, 40.05)
Drug availability	58.0	(50.54, 66.02)
Cost	16.0	(11.00, 22.78)
**NCD risk factors and knowledge**		
Smoke tobacco	32.0	(26.88, 37.36)
Use extra salt for most/all meals	61.0	(55.32, 66.26)
Know how to prevent or control diabetes	33.0	(28.05, 38.60)
Have heard a message about asthma or emphysema	63.0	(57.20, 68.06)

Abbreviations: CI, confidence interval; LBP, Lebanese Pound; NCD, noncommunicable disease; PHC, primary health care; SD, standard deviation.

All data reported as % (95% CI) unless otherwise specified.

^a^ Among respondents who have been to a PHC facility.

**TABLE 3.** Knowledge, Practice, and Coverage Survey of Syrian Refugee Mothers of Children Under 2 Years of Age in Lebanon, 2016 (N=300)

	%	95% CI
**Demographics**		
Mothers over 25 years old	55.5	(49.83, 60.99)
Mothers who have no education	40.3	(36.65, 46.08)
Mothers who completed primary school	20.5	(16.03, 25.50)
Mothers who completed secondary school	6.04	(3.62, 9.38)
**Breastfeeding**		
Children under 2 ever breastfed	90.0	(85.71, 92.98)
Infants 0–5 months exclusively breastfed	3.0	(1.20, 6.40)
Mothers of infants 0–5 months who gave their infant water	92.0	(87.49, 95.12)
Mothers of infants 0–5 months who gave their infant formula	44.0	(37.07, 50.49)
Mothers of infants 6–9 months who gave their infant breast milk and complementary foods	81.0	(69.09, 89.75)
Mothers of children 0–23 months who continue to breastfeed their infant aged 6–11 months	81.5	(71.30, 89.25)
Mothers of children 0–23 months who continue to breastfeed their child aged 12–17 months	54.0	(40.75, 67.28)
Mothers of children 0–23 months who continue to breastfeed their child aged 18–23 months	27.0	(17.20, 39.10)
**Child vaccination**		
Children 0–23 months who currently have a vaccination card (Child Health Card) (verified)	50.0	(41.45, 59.31)
Children 12–23 months who received a DPT1, DPT3, and measles vaccine, as verified by a vaccination card	100.0	(100.00, 100.00)
Children 12–23 months who received a DPT3 vaccine, as verified by a vaccination card, by 12 months of age	24.0	(16.95, 32.34)
**Water and sanitation**		
Households with an improved source for drinking water	90.0	(86.51, 93.21)
Households using an improved toilet facility	93.0	(89.51, 95.39)
Households with soap at a place for washing hands	100.0	(100.0, 100.0)
Mothers of children 0–23 months who washed their hands with soap at ≥2 of the appropriate times	65.0	(59.23, 69.96)
**Diarrhea**		
Mothers of children 0–23 months who report that their child had a diarrhea episode in the 2 weeks prior to the survey	55.0	(49.34, 60.53)
Children with a diarrhea episode treated with ORS	57.6	(49.65, 65.22)
Children with a diarrhea episode treated with more fluids	56.0	(47.83, 63.47)
Children with a diarrhea episode offered the same amount or more food	39.0	(31.31, 46.67)
**ARI**		
Mothers of children 0–23 months who report that their child had a cough and difficulty breathing/fast breaths in the 2 weeks prior to the survey	30.0	(25.32, 35.64)
Children 0–23 months with ARI in the last 2 weeks who were taken to an appropriate health care provider	37.0	(27.44, 48.13)
Children 0–23 months with ARI in the last 2 weeks who were taken to an appropriate health care provider within 2 days	19.0	(11.28, 28.22)

Abbreviations: ARI, acute respiratory infections; CI, confidence interval; DPT1, first diphtheria, pertussis, and tetanus (DPT) vaccine dose; DPT2, second DPT vaccine dose; DPT3, third DPT vaccine dose; ORS, oral rehydration solution; SD, standard deviation.

### Health Systems Strengthening Phase

With this evidence, in 2016, most health agencies including ours began following new guidance from the Lebanon Crisis Response Plan^35^—produced by the Government of Lebanon and the United Nations—to transition from mobile medical clinics to more sustainable forms of health systems strengthening. We initiated support for a PHC facility operated by a local NGO in Central Bekaa, through subsidized consultations and diagnostic tests for Syrian refugees and low-income Lebanese; quality improvement through continuous monitoring and staff supervision and training; and linkages to community outreach and referrals through refugee outreach volunteers (ROVs).

ROVs are a community-based health workforce for refugee communities endorsed by UNHCR, with standard terms of reference. We adapted this model to mobilize refugees in ISs who demonstrated an initiative to help their community, thereby developing health reference points in the settlements. We identified potential ROVs by selecting those who previously volunteered to participate in mobile clinic services, screening them first for literacy and interest, and then on NCD and technical knowledge.

Many settlements consist of large extended families, giving ROVs a starting advantage in recognition and authority. There is now at least 1 ROV per IS, and up to 5 for larger settlements. One or 2 of the most active ROVs per settlement were further selected as leaders through knowledge testing. Out of an initial recruitment of 500 ROVs, 120 leaders were selected to be the main focal points for health in the ISs, with women making up 90% of the cohort. While this may appear to be a gender imbalance, it does reflect the larger proportion of female refugees overall and the distribution of NCD diagnoses treated in the mobile clinics—two-thirds of our patients with chronic respiratory disease, type-2 diabetes, and hypertension are women. Due to cultural norms, female ROVs are also uniquely able to facilitate discussions on recently added women's health, antenatal care, and postnatal care topics.

Out of an initial recruitment of 500 ROVs, 120 leaders were selected to be the main focal points for health in the informal settlements, with women making up 90% of the cohort.

Once selected, ROVs were equipped with blood pressure cuffs and glucometers, and trained to monitor disease control. Training was delivered through participatory adult education sessions, led by our 5 staff community health promoters (CHPs), which covered disease knowledge basics and provided hands-on equipment demonstrations. Subsequently, ROVs worked alongside the CHPs in mobile clinics to learn how to measure blood pressure and glucose and how health education messages should be shared. Over the course of the following year, as their skills improved and refugee communities developed further trust in the system, ROVs became more independent and capable of monitoring NCD disease control on their own; they are now responsible for following patients with hypertension and diabetes every month in their own communities. CHPs and ROVs also map the epidemiology of NCDs in their communities through screening activities and individual cardiovascular disease risk stratification with a non-laboratory based method.^36^ The National Health and Nutrition Examination Surveys (NHANES) I Follow-up Study tool classifies future risk based on gender, diabetes status, tobacco use, blood pressure, body mass index (BMI), and age. ROVs measure blood pressure and calculate BMI during household visits or group meetings, and refugees classified as having high cardiovascular risk are referred to a PHC facility for specialized attention and follow up for intensive behavior modification.

### Community Health Methodologies

Using a social and behavior change framework, our staff CHPs facilitate behavior change sessions directly with refugees, train ROVs on priority health topics, and support them to act as referral and resource links between their communities and the health system. During monthly site visits, CHPs gather ROVs and other interested camp members for a participatory health education session on the topic of the month, usually meeting in a circle on the tent floor of the ROV leader or in a common area outside. The ROVs are given a pre- and post-test, and are tracked for attendance. Training methods incorporate strategies from the “Make Me a Change Agent” curriculum,^37^ such as effective communication, negotiation, guided testimonials, storytelling, and group facilitation. CHPs use large flip charts for interactive drawing activities or posters that illustrate the key points of the session. After being trained as trainers, ROVs are equipped with job aids, such as brochures or flip charts, and begin by co-facilitating health education sessions for other refugees in the IS. These sessions are supervised by CHPs who use quality improvement verification checklists to provide specific feedback to the ROVs. In this way, the ROVs are progressively developed as health leaders with ownership for outcomes in their own settlement.

Training methods for ROVs incorporate strategies from the “Make Me a Change Agent” curriculum, such as effective communication, negotiation, guided testimonials, storytelling, and group facilitation.

Because of the important connections between modifiable risk factors for NCDs, behavior change communication tools were adapted from International Federation of the Red Cross and Red Crescent materials^38^ to promote healthy lifestyle choices through participatory adult health education. Using these tools, CHPs and ROVs facilitate discussions and testimonials about realistic diet choices, such as avoiding high-fat, high-salt prepared foods, limiting sugar in tea and coffee, and reducing tobacco use. After our 2016 assessment and survey revealed additional gaps in women's and child health indicators, we adapted other health education materials to address yeast infections, skin diseases, and childhood pneumonia and diarrhea. Groups of pregnant women also gathered monthly to discuss antenatal and postnatal care and to monitor attendance at the PHC facility. In 2017, we began a collaboration with other NGOs to train specialized ROVs in basic mental health support—including group sessions on common concerns such as insomnia, psychosomatic symptoms, and child bedwetting—as well as early identification and referral for more complex psychological needs. This responsiveness to the changing priorities of beneficiaries has been an important element in building trust and partnership with refugee communities. We plan to undertake annual KPC surveys to monitor the impact of this behavior change strategy on indicators included in our baseline survey, such as tobacco use and changes in diet, and are currently using Barrier Analysis^39^ techniques to explore specific factors that allow some people to succeed in changing their behaviors.

In 2017, in collaboration with other NGOs, we trained ROVs in basic mental health support, including group sessions on common concerns such as insomnia, psychosomatic symptoms, and child bedwetting.

ROVs are also offered training opportunities from UNHCR and NGOs on relevant topics, such as referral mechanisms to PHC facilities or secondary care, breastfeeding, and mental health. Our staff facilitate twice-yearly visits for all ROVs to the PHC facility, to increase their visibility and connection to the health system. Throughout the year, the volunteers are also connected to supervisors and each other through a WhatsApp group on their mobile phones. Non-monetary incentives are given to ROVs, including bags, coats, mobile phone cards, and badges. However, unlike other ROV projects in the region, no monetary stipend is provided. This measure was taken to increase local ownership and sustainability as the crisis appears likely to continue for years.

### Project Health Information Systems

Project data collection forms are provided to each ROV to document health education target groups and participant numbers, health message content, referrals, blood pressure or sugar records, and NCD medication adherence. Each month, CHPs collect and aggregate the data on the ROV forms. In the first 2 months after the training and equipping phase by CHPs, ROVs in 80 settlements conducted 753 blood pressure monitoring visits for refugees with hypertension and 657 blood glucose checks for those with diabetes; monitored the medication adherence of 387 patients with NCD; referred 293 refugees to the local PHC facility for additional care for a wide range of conditions; and provided 346 targeted health education messages covering diarrhea, pneumonia, breastfeeding, and NCD topics (Table 4). Further impact data will be published in the future when the follow-up survey data are available.

**TABLE 4.** Refugee Outreach Volunteer Activities in Lebanon During Initial 2 Months of New Outreach Phase, 2016

	Blood Pressure Monitoring Visits	Capillary GlucoseMonitoring Visits	NCD Patient Medication Monitoring Visits	Refugees Referred to PHC Facility	Home Visits for Health Education
Total number	753	657	387	293	346
Monthly number per IS, mean (SD)	7.5 (2.9)	6.4 (3.1)	3.7 (1.8)	2.2 (1.1)	2.9 (1.6)

Abbreviations: IS, informal settlements; NCD, noncommunicable disease; PHC, primary health care; SD, standard deviation.

Accountability for refugee services is managed by uploading aggregated reporting from the NGOs to UNHCR's ActivityInfo platform on a monthly basis. Our mobile medical clinics initially used a paper-based health record system in the field, after which selected patient data were then consolidated into a computer database. This process required maintaining a large repository of paper records and many hours of transcribing information into a computer database. To simplify this process, we adapted the open-source mobile application CommCare (Dimagi, Cambridge, MA, USA) to create a health information system for refugees receiving NCD services. With consent, each patient was registered in the application on an Android tablet and the information stored in encrypted format and transmitted securely to a Health Insurance Portability and Accountability Act (HIPAA)-compliant central server when data connectivity was available. After registration, clinical data was recorded in linked forms, with diagnoses and medications stored and automatically populated in the digital file on each follow-up visit. Automatically aggregated data was then reported to UNHCR, as required. With the transition from mobile health clinics to community outreach, the application has been modified to collect information about CHP and ROV activities, mainly on antenatal and postnatal care. Because the application was free to deploy and can track cases over time, other health actors have expressed interest in adapting it as well. At the PHC level, we also facilitated the implementation of the national health information system software in order to integrate data collection with the Ministry of Health's network of public health facilities.

## DISCUSSION

Noncommunicable diseases are an increasing burden worldwide,^40^ particularly for Syrian refugees and the eastern Mediterranean region as a whole.^41^ With displacement at an all-time high, NCDs are an increasingly important issue in humanitarian health. In this article, we demonstrate that NCDs can be addressed alongside other common acute conditions in Syrian refugee settlements, through community health outreach, behavior change promotion, disease monitoring, and linkages to ongoing clinical care. Initially, our paid staff delivered health education in parallel to direct services in mobile clinics, but follow-up surveys showed gaps in behaviors and knowledge, which prompted an increased focus on community outreach for social and behavior change. While others have also made efforts to conduct NCD awareness sessions using paid health educators,^42^ we trained and equipped refugee volunteers with tools to facilitate changes in NCD-related behaviors and consistently monitor disease control. Early results are encouraging, with highly active ROVs gaining confidence in facilitating behavior change sessions, monitoring disease control, and making referrals.

We also found that participatory assessment and statistical survey methods could be adapted to the refugee context to guide program decisions and empower displaced people to set their own health priorities. Mobile health applications eased the burden of project data collection, improved reporting efficiency, and assisted in identifying long-term disease control trends. We are now applying these approaches to maternal, child, and mental health conditions in the same settlements alongside other health partners in Lebanon, an effort which should be explored and encouraged in other refugee contexts as well.

Participatory assessment and statistical survey methods can be adapted to the refugee context to guide program decisions and empower displaced people to set their own health priorities.

The successful provision of health services to vulnerable Lebanese and Syrian populations will require support for the public health system, coupled with a strong community presence and the task shifting of basic services to a community-based health workforce. The fragmented and highly privatized Lebanese health care system has historically focused on facility-based interventions, with limited community outreach. Prior to the refugee crisis, mechanisms for community-based health promotion, disease prevention, and linkages to the public health system were undeveloped and the use of community volunteers was rare.^43^ Given the expectation that Lebanon may host Syrian refugees for years to come, access to equitable health care for all vulnerable populations is vital to the economic and political well-being of Lebanon, and will require health outreach programs to link communities to existing services. In a recent hopeful sign for scaling up this approach, UNICEF's proposed “THRIVE Initiative” contains a 3-year US$21 million package for integrating community outreach into a child survival strategy for Lebanon.

## CONCLUSION

This article demonstrates one way humanitarian health programs can transition from direct service delivery into national health systems strengthening and community outreach. Refugees are capable and invested in serving their own communities, and can be effectively supported to facilitate NCD prevention, health promotion, and referrals to primary health care facilities. As global health experience has shown in development settings, mobilizing communities to participate in their own health outreach is highly effective and crucial for sustainable outcomes, a lesson that now can be transferred to protracted refugee crises.

Refugees are capable and invested in serving their own communities, and can be effectively supported to facilitate NCD prevention, health promotion, and referrals to primary health care.

## Notes

**Peer Reviewed**

**First Published Online:** 2017 September 19
